# Memory compromise at extended delays in people with new‐onset epilepsy

**DOI:** 10.1002/epi4.13022

**Published:** 2024-08-13

**Authors:** Remy Pugh, David N. Vaughan, Graeme D. Jackson, Jennie Ponsford, Chris Tailby

**Affiliations:** ^1^ School of Psychological Sciences Monash University Clayton Victoria Australia; ^2^ Florey Institute of Neuroscience and Mental Health Heidelberg Victoria Australia; ^3^ Department of Neurology Austin Health Heidelberg Victoria Australia; ^4^ Monash Epworth Rehabilitation Research Centre Epworth Healthcare Melbourne Victoria Australia; ^5^ Department of Clinical Neuropsychology Austin Health Heidelberg Victoria Australia

**Keywords:** ALF, cognition, epilepsy, memory, telehealth

## Abstract

**Objective:**

Memory is one of the most sensitive markers of cognitive compromise in people with new‐onset epilepsy. Nonetheless, around half of these cases score within the normal range on standard memory testing. Here we explore whether memory retention at a 1‐week delay reveals otherwise undetected memory compromise in such individuals, and how it relates to subjective memory complaints and mood.

**Methods:**

Using a prospective design, 38 adults with new‐onset epilepsy underwent baseline memory screening via telephone using an abbreviated Rey Auditory Verbal Learning Test (RAVLT). Psychological screening occurred via online questionnaires. One week later, without forewarning, participants completed three follow‐up memory tasks. Of particular focus, we explored longer‐term memory performances and forgetting trajectories in those individuals (*n* = 23) who demonstrated normal memory performances (scores >10th percentile) at baseline (30‐min delay). Outcomes were compared to 32 healthy controls, matched for age, sex, and education.

**Results:**

As a group, people with epilepsy performed worse than controls on all memory measures, with 44 percent impaired at baseline testing. Of those *unimpaired* at baseline, the rate and volume of information loss over 1 week was significantly greater than for controls. Contextual memory performance at 1 week was also significantly poorer for people with epilepsy. At the individual level, the prevalence of impaired forgetting was not significantly different between patients and controls. Subjective memory complaints were not related to any objective tests but were strongly related to self‐reported mood and anxiety symptoms.

**Significance:**

People with new‐onset epilepsy show reduced memory at short and extended intervals. For those showing normal memory at baseline, information does appear to be forgotten more quickly than in healthy controls, though the effect is not large. The findings indicate that while extended delay memory testing is not essential in all new epilepsy cases, it could provide useful information for particular individuals.

**Plain Language Statement:**

Memory problems are common in people with epilepsy shortly after seizure onset, however, many individuals still show normal memory performances on standard neuropsychological testing. Through testing memory at an extended timepoint (1 week), our study found that on average, these individuals showed a slightly quicker rate of forgetting over a 1‐week period than people without a brain condition. Self‐reported memory complaints in people with new epilepsy were unrelated to their actual memory skills on testing at short and long timepoints but were rather linked to lower mood and quality of life.

**Key points**

- Anterograde memory deficits are present in people with new‐onset epilepsy at short and extended delays.
- A mild effect of ALF is present in people with new‐onset epilepsy who demonstrate normal memory performances on standard testing.
- Subjective memory complaints are not related to objective memory at short or extended delays but are related to mood and quality of life.
- While routine ALF testing is not essential in all new‐onset cases, it may be useful in particular clinical cases.

## 1 INTRODUCTION

Cognitive impairment is prevalent in adults with new‐onset epilepsy, with memory problems among the most commonly observed deficits on standard neuropsychological assessment.^1^, ^2^ Nonetheless, a substantial portion of these individuals—approximately half of cases—perform within the normal range on memory tests.^1^, ^2^, ^3^

There is some evidence from the chronic epilepsy literature that there is a cohort of patients who show normal memory performances at standard delay intervals (20–30 min), but who subsequently forget information more rapidly than matched healthy volunteers.^4^, ^5^, ^6^, ^7^ This phenomenon, often referred to as accelerated long‐term forgetting (ALF),^8^ originated in the transient epileptic amnesia (TEA) literature and has since been of growing interest in epilepsy.^9^, ^10^, ^11^ ALF has been found across chronic epilepsy syndromes, and is now considered a key aspect of the clinical profile.^4^, ^5^, ^7^, ^12^ While debates continue regarding its operationalization and measurement,^9^, ^12^, ^13^ a core principle is that assessing memory over extended intervals has the potential to identify meaningful mnestic compromise that can be missed on standard clinical assessment. Given the apparent vulnerability of memory in new‐onset epilepsy,^1^ this raises the question of whether new‐onset individuals with apparently ‘normal’ memory on standard testing may nonetheless harbor evidence of memory compromise when evaluated for ALF. Further, while subjective memory complaints have shown only weak correlations with objective memory performances in chronic epilepsy research, some have argued that they are more strongly linked to memory at extended delays (i.e., days to weeks).^14^, ^15^ In this way, ALF may better explain patients' subjective memory complaints, concerns of which are common among people with new‐onset epilepsy.^16^, ^17^ No studies to date have explored ALF this early in the disease course. Thus, there are strong clinical reasons for studying memory across extended delays in new‐onset epilepsy cases, as this may provide prognostic information while at the same time objectively addressing patient complaints. Moreover, assessing ALF in this cohort minimizes the potential confounds known to the chronic epilepsy literature, such as long seizure and treatment histories, which can influence neuropsychological results.

Using a telephone‐based approach, we aimed to explore memory over an extended interval in adults with recent onset epilepsy. It was expected that those who show apparently normal memory on baseline testing would exhibit accelerated forgetting across a 1‐week period relative to healthy controls. Finally, we sought to investigate links between long‐term memory performances, subjective memory complaints, mood, and quality of life in new‐onset epilepsy.

## 2 METHODS

### 2.1 Participants

The current prospective study forms part of a larger project investigating neuropsychological functioning in patients of a First Seizure Clinic (FSC), approved under the Austin Health Ethics Committee (HREC: 59148). See Pugh et al.^1^ for detailed methods. Briefly, participants were recruited via referrals to the Austin Hospital FSC for a first suspected seizure between February 2021 and December 2023. Baseline cognitive and psychological screening were completed prior to patients' initial clinical appointment at the FSC. Inclusion criteria included aged 18 years and older, English‐speaking, have not been on antiseizure (ASM) for longer than 6 months. Data were collected on 46 individuals who were later diagnosed with epilepsy at the initial or subsequent FSC visit. Of these who underwent baseline cognitive screening, 43 completed the 1‐week follow‐up tasks and three were lost to follow up.

The control group comprised healthy volunteers who were identified as those responding to study flyers posted in community locations (supermarkets, universities, community notice boards) and on social media. Controls had no history of neurological condition, no current psychiatric condition, and who were not using psychotropic medication at time of testing. Of 60 who underwent neuropsychology screening, 58 completed the follow‐up memory tasks. In efforts to match controls to the epilepsy group for age, sex, and education, a subset of 32 controls were retained for the analysis based on demographic characteristics alone without consideration of neuropsychological scores. Controls completed cognitive and psychological screening in the same manner as patients.

### 2.2 Neuropsychological screening

Cognition was assessed via telephone by trained neuropsychologists using a standardized approach.^1^ Psychological screening occurred via online questionnaires administered by REDCap. The following measures were administered as part of our larger telephone screening battery:

At baseline:

- An abbreviated version of the Rey Auditory Verbal Learning Test (referred to hereafter as ‘RAVLT’), used to measure verbal learning and memory. This included three learning trials of 15 words^18^ followed by a delayed free recall trial after 20–30 min. Participants were not forewarned of delayed recall trials.
- Psychological measures included the Neurological Disorders Depression Inventory for Epilepsy (NDDI‐E),^19^ the Brief Epilepsy Anxiety Survey Instrument (brEASI),^20^ the Liverpool Adverse Event Profile (LAEP)^21^ and the Quality of Life in Epilepsy (QoLiE‐31).^22^ The latter tool was adapted to include experiences of ‘seizure(s) and/or epilepsy’ in place of ‘epilepsy’ on certain items, and the option of ‘N/A’ for items relating to ASM. A subjective memory score was also derived from four items drawn from the LAEP (1 item; “During the last 4 weeks, have you had any [memory problems]?”) and QoLIE‐31 (3 items; “In the past 4 weeks have you had any trouble with your memory?,” “How often in the past 4 weeks have you had trouble remembering things people tell you?,” “How much do your [memory problems] bother you on a scale of 1 to 5?”): a principal components analysis was run to reduce the dimensionality of the items, yielding an overall subjective memory score. The first component explained 85.58 percent of the variance, with greatest contributions from the LAEP “memory problems” item and QoLIE‐31 “have you had any trouble with your memory?”

One week after baseline screening (or as near to as possible), participants were called without forewarning that memory would be assessed and administered the below long‐term memory tasks via telephone. This timeframe has shown to be sensitive in previous literature.^23^

- RAVLT Free Recall trial in addition to a 30‐item Yes/No Recognition trial using an in‐house list comprising 15 target and 15 distractor items (scored as the discrimination index [DI] = true positives‐false positives).
- Additionally, we administered a measure of contextual incidental long‐term memory using a Memory for Tasks test, adapted from the ‘Memory in Reality’ paradigm of Helmstaedter et al.^24^ Several later studies have also found promising results when evaluating memory of real‐life events in epilepsy.^4^, ^25^, ^26^, ^27^ Our Memory for Tasks test was administered before the 1‐week delay RAVLT trials. It required participants to freely recall brief descriptions of each task they completed at baseline screening. One point was given for a correct and sufficient recollection of each task (i.e., a description that uniquely identified the main elements in a task) with a maximum score of 9 (full task list from the baseline assessment is described in Pugh et al.^1^). A single prompt for more information (“can you provide more detail?”) was provided for vague/ambiguous responses (e.g., “a task with numbers”). The Memory for Tasks test was introduced later into the protocol, and so data were only available for a subset of participants.

The actual interval between baseline and long‐delay memory testing ranged from 7 to 9 days for patients and 6 to 9 days for controls (see Table 1). The exception was two epilepsy cases who did not complete delayed memory testing until 21 and 22 days after baseline who were excluded from the analysis.

**TABLE 1:** Demographic characteristics by group.

	Epilepsy	Controls	*p* value
*N*	41	35	
Male/Female (*N*)	26/15	18/17	0.29
Age at testing (years)	47.36	45.21	0.64
Level of education			
≤12 years	24	14	0.29
Tertiary degree	14	17	
Post‐graduate degree	3	4	
Epilepsy diagnosis			
Focal	24		
Generalized	7		
Unknown	10		
Tonic clonic seizure, *N*	33		
Previous seizure history, *N*	14		
Presence of lesion on MRI, *N*	8		
On ASM, *N*	11		
Days between index event and cognitive screening, *Median* (range)	21 (2–132)		
Days between cognitive screening and 1‐week memory testing, *Median* (range)	7 (7–9)	7 (6–9)	

Abbreviations: ASM, antiseizure medication; MRI, magnetic resonance imaging.

### 2.3 Statistical analyses

Demographic characteristics of the sample were displayed in contingency tables and evaluated for significance using *t*‐tests for continuous variables and chi‐square for categorical variables. Group specific (Epilepsy, Control) means and standard deviations were calculated for all RAVLT variables and the Memory for Tasks Test (*n* = 62), and groups were compared using independent samples *t*‐tests.

Our main interest in this study was the subgroup of participants who showed ‘normal’ memory performances on the RAVLT 30‐min delay trial (‘baseline unimpaired’), which we defined as a *z*‐score greater than −1.28 (i.e., score above the 10th percentile). Focusing on this subgroup also minimized the potential of floor effects on the long‐term measures, an issue of concern in those with already poor baseline performances. To evaluate between‐group differences in the loss of information over time, we used a mixed ANCOVA with factors of *group* (Epilepsy, Control) and *time* (30‐min delay/T4 score, 1‐week delay/T5 score), using raw trial scores as the dependent variable. The last learning trial score (RAVLT T3) was used as a covariate, capturing whether the number of words lost was proportional to initial learning. Under this model, a significant group*time interaction indicated a difference between groups in the rate of information lost over time (ALF). To explore group differences in the percentage of information retained between 30‐min and 1 week, we performed a one‐way between groups ANOVA, using the percentage retained score ([T5 score/T4 score] × 100) as the dependent variable.

The frequency of ALF in our cohort was determined as the proportion of participants showing an impaired raw/percentage loss score over 1‐week relative to control scores (*z* < −1.28, <10th percentile). Impairment rates were statistically compared between groups (Epilepsy, Control) using chi‐square tests or Fisher's Exact tests where a cell count was less than 5.

The Memory for Tasks test and RAVLT Recognition DI were only obtained at the 1‐week delay (not at baseline also). Group differences on these measures for the *baseline unimpaired* subgroup were analyzed using independent samples *t*‐tests.

To explore potential factors associated with longer‐term memory in all participants with epilepsy, Pearson correlations were performed between the raw 1‐week memory measures (RAVLT Free Recall score, RAVLT Recognition DI, Memory for Tasks Test) and (i) subjective memory score, (ii) mood and anxiety symptoms (on the NDDI‐E and brEASI, respectively), and (iii) quality of life (QoLiE‐31).

All analyses were carried out using R/R Studio. The significance level was set at *p* < 0.05.

## 3 RESULTS

### 3.1 Whole sample

#### 3.1.1 Sample characteristics

Table 1 displays the sample characteristics for the epilepsy and control groups, respectively. The groups did not differ significantly on age, sex or level or education (*p* > 0.05). Of the epilepsy group, 24 received a diagnosis of focal epilepsy, 7 of generalized epilepsy, and in 10 the type was unknown. Within the focal cases, 8 had a suspected temporal focus, 4 an extra temporal focus and 12 were unknown. For the majority, the index event was a tonic clonic seizure. Of 14 (34%) who had described a history of probable seizures prior to the index, four participants reported one prior seizure, five reported two to three events, and five reported three or more past seizures. Only 8 of 39 for whom MRI data was available had evidence of an epileptogenic lesion. Baseline screening occurred at least 2 days (no less than 48 h) after the most recent seizure.

A minority of the epilepsy group were on ASM at the time of initial screening (11 of 41), all of whom were on monotherapy. In a supplementary multivariate analysis of variance (MANOVA), we found no significant differences on the combined raw RAVLT variables between participants who were taking and not taking ASM at the time of testing (*F*(1, 39) =0.58, *p* = 0.68). We therefore included both those who were medication naïve and those who had recently commenced ASM in the epilepsy group.

#### 3.1.2 Performances across all memory measures by group

As seen in Table 2, the entire epilepsy group (*n* = 41) performed significantly worse than controls on all raw scores of the RAVLT, including Total Learning, 30‐min Delay, 1‐week Free Recall, and Recognition DI (*p* < 0.05). For participants with Memory for Tasks Test data (*n* epilepsy = 32, *n* control = 30; remaining matched for age [*p* = 0.44], sex [*p* = 0.83] and education [*p* = 0.21]), the epilepsy group recalled significantly fewer tasks at 1‐week than controls (*p* < 0.001). Of note, nine people with epilepsy and two controls were at the floor on the RAVLT 1‐week Free Recall and, therefore, memory loss may be underestimated in these cases. This issue was mitigated in our primary analyses, presented below, focussing on the *baseline unimpaired* group.

**TABLE 2:** Learning and memory raw scores for the whole sample of patients and controls.

Measure	Epilepsy (*n* = 41), *M* (SD)	Control (*n* = 35), *M* (SD)	*p* Value	Cohen's *d*
RAVLT learning total (T1‐3)	20.93 (5.60)	28.31 (4.83)	<0.001	1.40
RAVLT 30‐min recall (T4)	5.51 (2.97)	8.49 (2.95)	<0.001	1.00
RAVLT 1‐week recall (T5)	2.15 (1.80)	5.03 (2.72)	<0.001	1.27
RAVLT 1‐week recognition DI	6.66 (3.31)	9.91 (3.18)	<0.001	1.00
Memory for Tasks test^a^	3.44 (1.68)	4.97 (1.30)	<0.01	1.01

Abbreviations: DI, Discrimination Index; RAVLT, Rey Auditory Verbal Learning Test.

^a^ Epilepsy *n* = 32, Control *n* = 30.

#### 3.1.3 Prevalence of memory impairment at baseline (30‐min delay)

Table 3 displays the frequency of memory impairment on the RAVLT 30‐min delay relative to the healthy controls. Forty‐four percent of the epilepsy group (18 of 41) showed impaired performances (*z* < −1.28; <10th percentile) compared to nine percent of controls (3 of 35). The following analyses focus on those patients (*n* = 23) and controls (*n* = 32) who were unimpaired (*z* ≥ −1.28; ≥ 10th percentile) on the RAVLT 30‐min delay trial at baseline memory testing.

**TABLE 3:** Frequency of memory impairment on the RAVLT 30‐min delay by group.

Group	Unimpaired, *N*	Impaired, *N*	% Impaired
Epilepsy	23	18	44
Control	32	3	9

*Note*: Memory impairment based on *z*‐score < −1.28; <10th percentile.

### 3.2 Baseline unimpaired sample

#### 3.2.1 Rate of memory decay (ALF) over 1‐week

**FIGURE 1:**
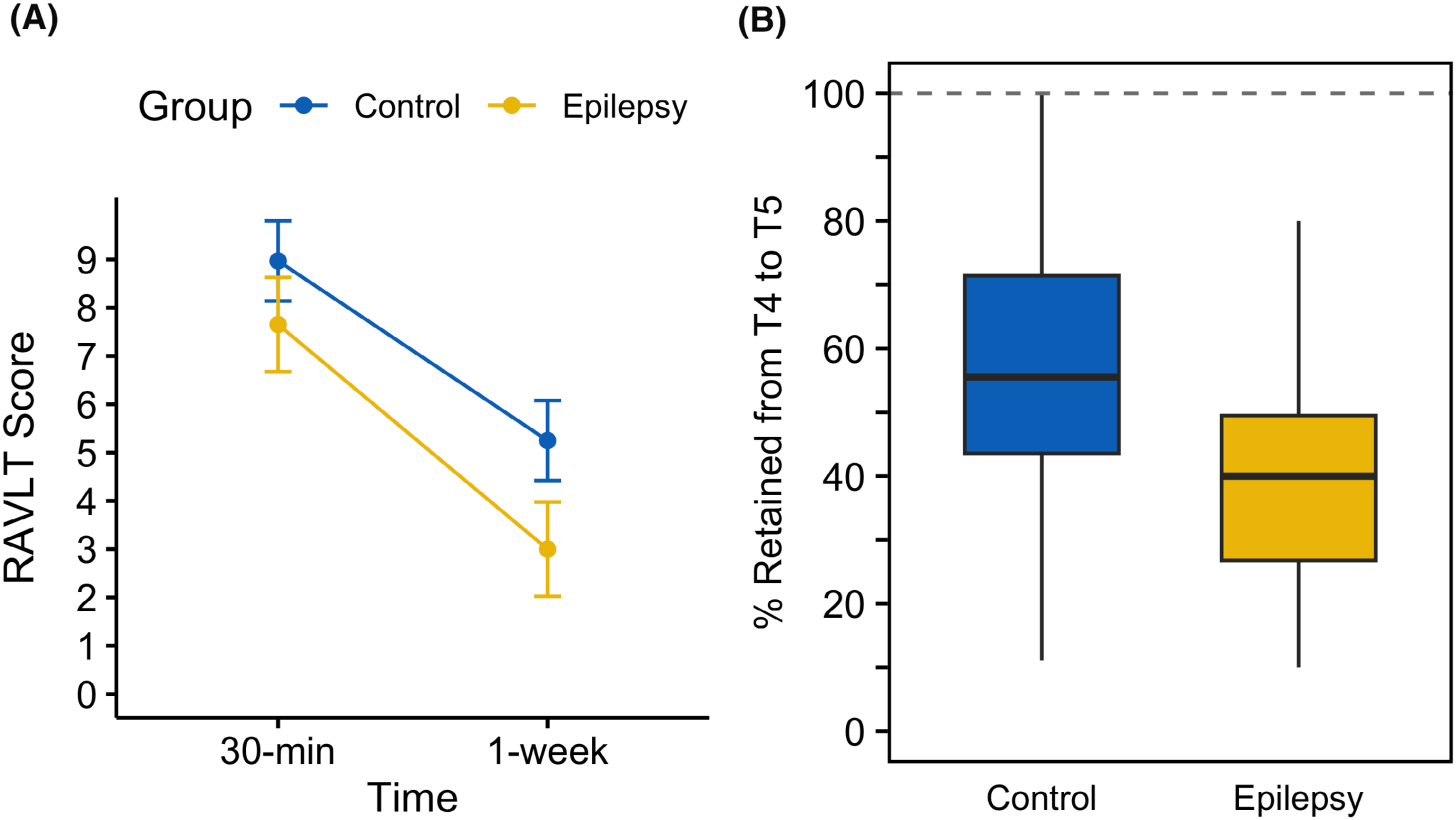
Memory decay on the RAVLT between 30‐min and 1 week for the baseline unimpaired subgroup by group, denoted by the raw loss of words (A) and the percentage of information retained over time (B). Error bars in (A) reflect 95% lower‐confidence intervals. Dashed horizontal line in (B) denotes 100% retention (i.e. no memory decay across the week). RAVLT, Rey Auditory Verbal Learning Task.

Regarding the total number of words recalled on the RAVLT, the *baseline unimpaired* epilepsy group recalled slightly fewer words at the 30‐min delay than controls (*p* < 0.05, Table 4), and there was a significant group‐by‐time interaction (*F*[1,52] = 5.92, *p* = 0.02, $\eta_{p}^{2}$ = 0.10) with the epilepsy group losing words at a faster rate between the 30‐min and 1‐week delay trials (see Figure 1A). Significant differences were also found in the percentage of information retained over 1 week (*F*[1, 53] = 8.95, *p* = 0.004), with the epilepsy group retaining less information over 1 week (39%) compared to controls (58%; Figure 1B). Thus, the baseline unimpaired epilepsy group exhibited lower scores at 30‐min than baseline unimpaired controls, and the amount of information lost across 1 week was greater in the epilepsy group, measured either as total number or percentage of words lost.

**TABLE 4:** Learning and memory raw scores for the ‘baseline unimpaired’ participants by group.

Measure	Epilepsy (*n* = 23), *M* (SD)	Control (*n* = 32), *M* (SD)	*p* Value
RAVLT learning total (T1‐3)	22.96 (5.93)	29.09 (4.25)	<0.001
RAVLT 30‐min delay (T4)	7.65 (2.04)	8.97 (2.60)	0.04
RAVLT 1‐week free recall (T5)	3.00 (1.68)	5.25 (2.72)	<0.001
RAVLT 1‐week recognition DI	8.48 (2.71)	10.22 (2.92)	0.02
Memory for Tasks test	3.76 (1.64)	5.00 (1.30)	0.01

Abbreviations: DI, Discrimination Index; RAVLT, Rey Auditory Verbal Learning Test.

#### 3.2.2 Prevalence of ALF

Regarding the raw loss of words over 1 week, rates of impairment (defined as *z* < −1.28) were comparable between the groups, with 18 percent (4 of 23) of the epilepsy group exhibiting an impaired change score compared with 12.5 percent (4 of 32) of controls (*p* > 0.05). Similarly, when expressed as the percentage of information lost, 26 percent (6 of 23) of the epilepsy group showed an impaired change score relative to 12.5 percent (4 of 32) of controls (*p* > 0.05).

#### 3.2.3 Long‐term memory performance for contextual information and recognition memory

Within the *baseline unimpaired* sample, the epilepsy group performed significantly poorer than controls on the RAVLT Recognition DI (*p* = 0.02) and the Memory for Tasks test at 1 week (Epilepsy *N* = 17, Control *N* = 27; *p* = 0.01; Table 4).

### 3.3 Relationships between measures of long‐term memory, subjective memory complaints, and psychological factors for the whole epilepsy group

**FIGURE 2:**
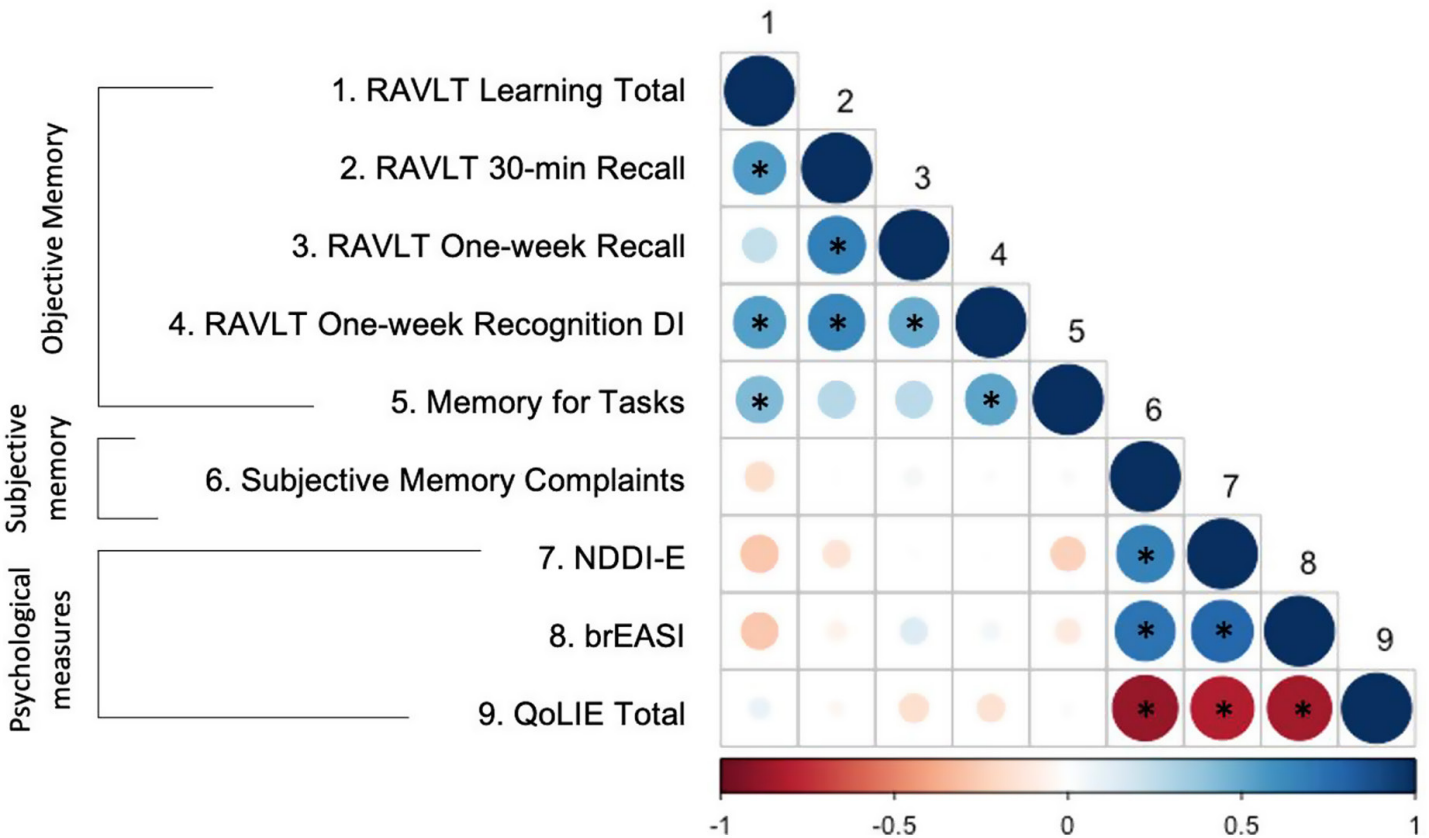
Correlations between objective memory variables, subjective memory, mood, and quality of life for all people with epilepsy. *Significant at *p* < 0.05. brEASI, brief Epilepsy Anxiety Survey Instrument; DI, discrimination index; NDDI‐E, Neurological Disorders Depression Inventory for Epilepsy; RAVLT, Rey Auditory Verbal Learning Task.

To explore factors associated with extended delay memory performance in all participants with epilepsy, correlations between raw objective memory scores, subjective memory complaints, and psychological variables were calculated and displayed in Figure 2. Of relevance, subjective memory complaints were not significantly associated with any objective 1‐week delay memory measures (*p* > 0.05). Subjective memory complaints, however, were strongly positively correlated with depression (*r* = 0.66, *p* < 0.001, *M* = 12.81, *SD* = 4.44) and anxiety scores (*r* = 0.73, *p* < 0.001), as well as poorer quality of life (*r* = −0.87, *p* < 0.001, *M* = 5.11, *SD* = 5.92). No objective measures of 1‐week delay memory (RAVLT 1‐week Recall, RAVLT Recognition, Memory for Tasks) were correlated with depression, anxiety scores, or quality of life scores (*p* > 0.05). The same pattern of results was found when examining just the ‘baseline unimpaired’ group.

In exploring the relationships between long‐term memory measures, RAVLT 1‐week Recall was significantly correlated with RAVLT Recognition DI (*r* = 0.50, *p* = 0.001), but not Memory for Tasks (*r* = 0.27, *p* > 0.05). Memory for Tasks performance was significantly related to RAVLT Recognition DI at 1 week (*r* = 0.53, *p* = 0.003).

## 4 DISCUSSION

This study explored extended delay memory function people with new‐onset epilepsy. In particular, we predicted that those with normal memory performances on standard within‐session testing would show ALF over a week relative to healthy controls.

ALF, as traditionally understood, captures memory deficits that only become apparent with longer‐term testing following baseline performances that are matched to controls.^8^, ^10^ In contrast, we showed that as a group, people with new‐onset epilepsy exhibited reduced memory at all time intervals, a finding that is common in other chronic epilepsy studies,^5^, ^6^, ^7^ with almost half impaired at 30‐min. Even considering only those patients who performed within ‘normal’ limits at 30‐min (>10th percentile), this subset still scored lower than controls at this timepoint. Nonetheless, in these individuals, we observed a subtle yet meaningful effect of ALF over a week, such that they lost more information over this interval than controls. It is worth mentioning that the results reflect an average rate of forgetting over a 1‐week period. The exact and possibly dynamic rate of information decay between 30‐min and 1 week for either group cannot be known.

Overall, our data suggest a mild degree of ALF in new‐onset epilepsy, apparent on the RAVLT. To date, the exploration of ALF in epilepsy has been limited to chronic disease cohorts (mainly TLE), in which studies have found varying degrees of its presence. Findings among ALF studies are greatly influenced by measurement choice (e.g., measure selection, follow‐up timeframes, definition of ALF, impairment cut scores, binary versus continuous outcome),^12^, ^13^ which hampers the comparison of results between studies. Nonetheless, it appears that a greater degree of ALF is present in those with hippocampal lesions,^12^, ^26^, ^28^, ^29^ limbic encephalitis,^13^ and a left‐lateralized seizure focus.^9^, ^13^, ^26^, ^27^ Therefore, the fact that ALF was not as strongly observed in our heterogenous sample likely relates to the epileptological attributes of our cohort, being predominantly non‐lesional, unmedicated epilepsy with a single seizure. While the above features—particularly hippocampal insult—appear to be risk factors for strong ALF effects (at least in chronic epilepsy) they are not sufficient nor required to produce ALF,^6^, ^13^, ^27^, ^28^ suggesting that other factors are also at play. There are fewer studies examining ALF in non‐TLE cohorts. One such study in people with chronic generalized epilepsy observed similar forgetting trajectories to ours over a 4‐week period on the RAVLT,^7^ though this pattern is not observed in other studies of generalized epilepsy.^30^ This may relate to methodological variability across studies of ALF as discussed above.

In addition, we also found that people with new‐onset epilepsy showed significantly poorer recall of contextual information after 1 week relative to controls, regardless of whether their memory at baseline (on the RAVLT) was impaired or not. Reduced memory of contextual information has also been documented in adults with TLE over 3^4^ and 4 weeks,^5^, ^26^ in those with TEA over 1 and 3 weeks,^31^ and in heterogenous samples of people with chronic epilepsy over 72 h^27^ and 1 week.^32^ Ricci et al.^25^ found that patients without hippocampal lesions (including ETE; *n* = 11) showed ALF for prose information between 1 and 4 days, only if information was learnt‐to‐criterion, whereas for TLE patients with hippocampal lesions (*n* = 12), memory decay occurred most rapidly during the first 24‐h after which forgetting plateaued. Interestingly, these groups performed similarly at the 4‐day delay, and differences in forgetting trajectories were only revealed through testing memory at several timepoints (30 min, 24 h and 4 days). Conversely, studies in adults with generalized epilepsies have found no evidence of ALF for contextual information over a 1‐^30^ and 3‐week period,^29^ although both indicated poor initial learning of information. Taken together, the findings again suggest that ALF may be weaker in non‐lesional, non‐TLE cohorts.

Historically, the underpinnings of ALF in epilepsy were thought to reflect two differentially affected memory systems, whereby early‐stage memory processing is relatively spared, after which the impaired, longer‐term (a.k.a. late) consolidation process causes rapid decay of memories. More recent discussions have highlighted the lack of empirical evidence for this theory or that forgetting accelerates after a specific time interval (e.g., more than 1 h or 1 week), arguing that memory decay in epilepsy is more likely a more fluid process.^9^ Specifically, a ‘slow‐to‐fast’ forgetting rate may reflect an initial compensation (synaptic or via conscious rehearsal) of a fundamentally disrupted memory system that inevitably emerges at longer intervals.^9^ By this account, the extended test interval allows a subtle underlying memory difficulty in these cases to become more apparent. Extending previous work, our findings of long‐term memory compromise cannot be attributable, exclusively, to the effects of macroscopic lesions (especially in the mesial temporal lobe), recurrent seizures,^7^, ^12^, ^27^, ^33^ ASM,^4^, ^7^, ^12^, ^27^, ^32^ chronicity of disease,^7^, ^33^ or mood.^4^, ^5^, ^15^ However, subclinical epileptic activity and/or underlying brain network disruption are possible bases for the memory difficulties observed in this cohort.^5^, ^15^, ^34^ These neural disturbances are apparently present at, or potentially before, disease onset.^1^, ^35^, ^36^, ^37^

While ALF was detectable at the group level, at the individual level, the prevalence of ALF (classified as present/absent) in our cohort was not significantly different from controls. As raised in the literature,^12^ the results can depend on the choice of cut score used (e.g., −1 vs. −1.5 SD). We observed similar variability in our data (not reported), however, the overall pattern indicated that ALF is not a particularly common or early marker of memory impairment in new‐onset epilepsy. Rather, verbal memory deficits were common and detectable at earlier (and all) timepoints. Therefore, the majority of new‐onset cases with objective memory deficits would be detected with standard memory testing, with only a small portion of individuals who show ALF ‘missed’ using standard neuropsychological testing. In this light, routine assessment of ALF appears unnecessary for all new cases of epilepsy. Standard within session assessment captures much of the information about mnestic function in this cohort as a whole. ALF testing may nonetheless be a useful additional tool in individual cases; where ALF is suspected based on the clinical history or with higher‐risk groups (e.g., hippocampal lesions, limbic encephalitis, left‐sided seizure focus), or where more evidence of memory performance is needed over longer intervals. Alternatively, findings could be used as a therapeutic tool to reassure the patient of a normal memory profile when tested at both short and longer delays.

There is some evidence that subjective memory complaints are more closely linked to longer‐term memory than that measured on standard testing in people with epilepsy.^14^, ^15^ In contrast, we found that no long‐term objective memory performances were associated with subjective complaints, nor with mood or anxiety symptoms, consistent with other studies.^4^, ^29^, ^30^ Rather, subjective complaints were strongly associated with higher anxiety and depression symptoms, and poorer quality of life, consistent with findings in chronic epilepsy^4^, ^30^, ^38^ and in new‐onset epilepsy.^16^, ^39^ In this regard, it is important that clinicians do not rely on self‐reported memory complaints as a means to infer actual memory function. Rather, subjective complaints may reflect changes in one's psychological state, or broader cognitive difficulties (i.e., in attention or processing speed) commonly interpreted by patients as memory problems.

Some limitations of the study should be noted. For screening purposes, we employed an abbreviated version of the RAVLT with fewer learning trials. This provided fewer learning opportunities (arguably more akin to everyday situations) and consequently has greater potential for floor effects at the follow‐up trials. We minimized this issue in our primary analyses by focusing on those with unimpaired baseline performances and controlling for last learning trial scores. However, it is possible that people with *impaired* memory at baseline also show ALF,^6^ yet this was not empirically testable due to floor effects. A list‐learning task with more learning trials may be beneficial in future studies. Secondly, although there were no significant differences between the epilepsy and controls groups in level of education or gender, slightly more of the controls were female or had tertiary degrees. While either of these factors may influence verbal memory performance, the demographic differences in our sample were not marked, suggesting that any such effects would be modest and unlikely to drive the results. Third, the sample size was relatively small, particularly for the ‘baseline unimpaired’ subgroup of people with epilepsy. In this regard, we were not in a position to compare findings between various clinical subgroups (epilepsy syndrome, seizure history or lesional status). Finally, prospective work with longitudinal assessment of ALF following epilepsy onset could help to determine the temporal progression of memory deficits over the disease course.

Overall, the present study found a high incidence of memory impairment in people with new‐onset epilepsy soon after the first seizure. A mild effect of ALF was able to be detected via telephone in those who did not show memory impairments on standard testing. While the proposed basis of long‐term memory compromise—including mild ALF—in new‐onset epilepsy remains unclear, our data suggest that recurrent seizures, ASMs and medial temporal pathology are not required for its emergence.

## AUTHOR CONTRIBUTIONS

**Remy Pugh:** conceptualization; project investigation and administration (lead); formal analysis (lead); writing – original draft (lead). **David N. Vaughan:** resources (equal); writing – review and editing (equal). **Graeme D. Jackson:** resources (equal); writing – review and editing (equal). **Jennie Ponsford:** supervision (supporting); writing – review and editing (equal). **Chris Tailby:** conceptualization; project administration; supervision (lead); writing – review and editing (lead).

## CONFLICT OF INTEREST STATEMENT

None of the authors has any conflict of interest to disclose. We confirm that we have read the Journal's position on issues involved in ethical publication and affirm that this report is consistent with those guidelines.
